# Study on intracranial meningioma using PET ligand investigation during follow-up over years (SIMPLIFY)

**DOI:** 10.1007/s00234-021-02683-1

**Published:** 2021-03-10

**Authors:** Hanne-Rinck Jeltema, Marnix R. Jansen, Adriaan R. E. Potgieser, Antoinette D. I. van Asselt, Mart A. A. M. Heesters, Anouk van de Hoorn, Andor W. J. M. Glaudemans, J. Marc C. van Dijk

**Affiliations:** 1 Department of Neurosurgery, University of Groningen, University Medical Center Groningen, Hanzeplein 1, P.O. Box 30.001, 9700RB, Groningen, the Netherlands; 2 Faculty of Medical Sciences, University of Groningen, University Medical Center Groningen, Groningen, the Netherlands; 3 Department of Epidemiology, University of Groningen, University Medical Center Groningen, Groningen, the Netherlands; 4 Department of Radiation Oncology, University of Groningen, University Medical Center Groningen, Groningen, the Netherlands; 5 Department of Radiology, University of Groningen, University Medical Center Groningen, Groningen, the Netherlands; 6 Department of Nuclear Medicine and Molecular Imaging, University of Groningen, University Medical Center Groningen, Groningen, the Netherlands

**Keywords:** Meningioma, ^11^C-Methionine PET, Positron emission tomography, Follow-up scanning, Stereotactic radiotherapy, SRT

## Abstract

**Purpose:**

Radiologic follow-up of patients with a meningioma at the skull base or near the venous sinuses with magnetic resonance imaging (MRI) after stereotactic radiotherapy (SRT) and neurosurgical resection(s) can be difficult to interpret. This study evaluates the addition of ^11^C-methionine positron emission tomography (MET-PET) to the regular MRI follow-up.

**Methods:**

This prospective pilot study included patients with predominantly WHO grade I meningiomas at the skull base or near large vascular structures. Previous SRT was part of their oncological treatment. A MET-PET in adjunct to their regular MRI follow-up was performed. The standardized uptake value (SUV) was determined for the tumor and the healthy brain, on the pre-SRT target delineation MET-PET and the follow-up MET-PET. Tumor-to-normal ratios were calculated, and ^11^C-methionine uptake over time was analyzed. Agreement between the combined MRI/MET-PET report and the MRI-only report was determined using Cohen’s κ.

**Results:**

Twenty patients with stable disease underwent an additional MET-PET, with a median follow-up of 84 months after SRT. Post-SRT SUV T/N ratios ranged between 2.16 and 3.17. When comparing the pre-SRT and the post-SRT MET-PET, five categories of SUV T/N ratios did not change significantly. Only the SUV_peak_ T/N_cortex_ decreased significantly from 2.57 (SD 1.02) to 2.20 (SD 0.87) [*p* = 0.004]. A κ of 0.77 was found, when comparing the MRI/MET-PET report to the MRI-only report, indicating no major change in interpretation of follow-up data.

**Conclusion:**

In this pilot study, ^11^C-methionine uptake remained remarkably high in meningiomas with long-term follow-up after SRT. Adding MET-PET to the regular MRI follow-up had no impact on the interpretation of follow-up imaging.

## Introduction

Meningiomas are frequently encountered intracranial tumors, with an annual incidence of 4–5 per 100,000. They compromise 20–30% of all intracranial tumors and have a predominance in female patients [1–3]. The majority of meningiomas is histopathologically benign (WHO grade I), with a smaller subset showing more aggressive behavior (WHO grades II and III) [3]. Disease control is dependent on the extent of resection, e.g., Simpson grade, and cytogenetic aspects of the tumor [2]. Gross total resection is an important factor to obtain disease control but is often difficult to achieve in meningiomas at the skull base or with a close relation to large vascular structures, e.g., a venous sinus. In the literature, recurrence rates up to 26.5% are encountered for skull base meningiomas [4, 5]. For parasagittal meningiomas, with close relation to the superior sagittal sinus, a recurrence rate of 16.7% is reported [6]. Hence, in case of meningiomas with a difficult anatomical localization, treatment is often multimodal with (repeated) neurosurgical resection(s) and various forms of radiotherapy [4].

Radiological follow-up of meningiomas after SRT, with or without neurosurgical resection(s), is challenging [7, 8]. Radiotherapy effects can mimic tumor progression. Vascular structures surrounding the tumor and surgical scar tissue hamper the interpretation of gadolinium enhancement on MRI. Whether an enlarging dural tail is a sign of recurrent tumor or a reactive phenomenon is difficult to distinguish by MRI. Also, meningioma infiltration in hyperostosis is difficult to discern on MRI.

The added value of nuclear imaging is that it provides information on the metabolic characteristics of a lesion. Fluorodeoxyglucose PET (FDG-PET) is probably the most often employed technique in oncology and is based on the elevated glucose uptake in a tumor. Since glucose metabolism in the brain is very high, it is not the preferred technique for nuclear imaging of intracranial lesions. However, PET based on amino acid metabolism, e.g., ^11^C-methionine PET (MET-PET) and ^18^F-fluoroethyl-L-tyrosine PET (FET-PET), harbors promising characteristics for intracranial use and is informative on cell metabolism and proliferation. As such, MET-PET has proven to be of great value for diagnostic purposes in multiple types of intracranial tumors [9, 10].

Current literature supports the use of MET-PET for the improvement of target delineation in SRT, in adjunct to the regular 3D planning MRI. The combination of the two imaging modalities results in a more accurate radiotherapy field and improved local disease control [11–13]. Ikeda et al. [14] advocated the role of MET-PET in the follow-up of meningiomas, but they included no patients with stereotactic irradiated meningiomas. The publication of Ryttlefors et al. on MET-PET as a follow-up technique in meningioma patients treated with proton beam therapy reported a significant decrease in ^11^C-methionine uptake during follow-up [15]. The availability of MET-PET, however, is limited to centers who have an on-site cyclotron at their disposal, for the production of this tracer with a relatively short half-life.

Regarding PET imaging for meningiomas, tracers targeted against the somatostatin receptor (e.g., SSTR2a), like ^68^Ga-DOTATATE and ^68^Ga-DOTATOC, have been described in the literature with promising results [16–18]. Galldiks et al. published an overview of the indications for ^68^Ga-DOTATOC and ^68^Ga-DOTATE PET in meningioma patients, according to the RANO/PET-group [7]. There is literature regarding the added value of ^68^Ga-DOTATATE/^68^Ga-DOTATOC PET for radiotherapy treatment planning [19–21]. There are a limited number of publications on the role of these type of PET tracers in the follow-up of meningiomas [22]. ^68^Ga-DOTATATE and ^68^Ga-DOTATOC PET are informative on receptor expression, while arguably amino acid PET tracers could be more informative about the metabolic state of the tumor.

MRI and PET are complementary diagnostic tools that provide structural and metabolic information, respectively^7^. In the University Medical Center Groningen, there is historically a long experience with amino acid PET tracers. Since MET-PET was used for radiotherapy treatment planning purposes in our center, we questioned if this amino acid tracer would also be beneficial in the follow-up of meningioma patients after SRT. In the current prospective study, we evaluated ^11^C-methionine uptake in meningiomas after SRT and explored the role of additional MET-PET to the regular MRI follow-up, based on the hypothesis that combined MET-PET/MRI should yield better information than MRI alone.

## Methods

### Ethical standard

The study protocol was approved by the local ethical committee (METc2017/572/ABR nr. NL63750.042.17). The study was conducted in accordance with the Declaration of Helsinki and its later amendments. Written informed consent was obtained from all patients prior to participation in the study.

### Patients

Patients with meningiomas at the skull base or near large vascular structures, previously treated with SRT using MET-PET in adjunct to the 3D planning MRI, were invited to participate in this study. Previous neurosurgical resection of the lesion was not an exclusion criterium. The study information was sent to 63 patients. Twenty-seven patients returned a signed informed consent form. Twenty patients underwent a MET-PET in adjunct to their regular follow-up MRI. Seven patients could eventually not participate for different logistical reasons (e.g., not enough radiolabeled tracer available on the day of examination; PET investigation could not be combined with MRI on the same day, which made the patient decide not to participate anymore; MRI follow-up interval of 2 or 3 years with no MRI planned during the research period).

### Magnetic resonance imaging

The regular follow-up MRI was performed on a 1.5T or 3T Siemens scanner combined with an 8-channel phased array head coil covering the whole head. The standard protocol consisted of a FLAIR, T2, DWI, SWI, T1 SE with and without contrast, and 3D T1 after contrast. The parameters of the standard protocol on 1.5T were for FLAIR [repetition time/echo time (TR/TE) 5000/335 ms; inversion time 1800 ms; voxel size 1.0 × 1.0 × 1.0 mm; no slice gap; flip angle 120°; number of excitations (NEX) = 1], T2 [TR/TE 4690/93; voxel size 0.6 × 0.45 × 5.0 mm; slice gap 0.5; flip angle 150° NEX = 2], SWI [TR/TE 49/40; voxel size 0.72 × 0.72 × 2.0; no slice gap; flip angle 15°], diffusion weighted imaging EPI sequence with accompanying ADC [*b*-value 0 and 1000; TR/TE 4400/98, voxel size 0.6 × 0.6 × 5 mm; slice gap 0.5 mm; flip angle 90°, NEX = 3], T1 spine echo before and after contrast [TR/TE 550/8.90; voxel size 0.7 × 0.7 × 5 mm; slice gap 0.5 mm; flip angle 90°, NEX = 1], and 3D T1 MPRAGE after contrast [TR/TE 9/4 ms; 1.0 × 1.0 × 1.0 mm; no slice gap; flip angle 8°; NEX = 1]. Intravenous contrast administration of 0.1 mmol/kg body weight gadopentetate dimeglumine [Dotarem] was used for the post-contrast images.

### ^11^C-Methionine positron emission tomography

Follow-up MET-PET scans were acquired on an integrated PET/CT camera system (Biograph mCT 40 or 64 slice PET/CT, Siemens, Knoxville, TN, USA) and conform EANM procedure guidelines for brain tumor imaging using labeled amino acid analogues [23]. Patients fasted for at least 6 hours before the intravenous injection of 200 MBq ^11^C-methionine. PET imaging over a period of 5 minutes was performed approximately 20 minutes after the administration of the tracer. Low-dose CT scan was performed for attenuation correction and anatomic mapping with 80 kV and 30 mAs.

### Measuring standardized uptake volumes

The ^11^C-methionine uptake in the stereotactically irradiated meningiomas was measured. Each tumor volume was captured in a 3D ellipsoid volume. SUV_max_, SUV_peak_, and SUV_mean_ were determined. Also, for the healthy contralateral side (mirror) and the healthy right parietal region (cortex), SUV_max_, SUV_peak_, and SUV_mean_ were determined. The right parietal area was chosen, because this is remote from most skull base meningiomas and possible adjacent diseased dura mater. Six different types of tumor-to-normal ratios (T/N ratios) were calculated, as is described in Table 1 (in which also a comparison to T/N calculation methodology in similar types of publications, cited in this work, is given).

**Table 1:** Overview of variance in the methodology used to calculate T/N ratios in MET-PET scanning in meningioma patients

Author	Method of calculating T/N ratio
Arita et al. (2012) [24]	SUV_mean_ tumor / SUV_mean_ normal cerebral cortex (no region specified) SUV_max_ tumor / SUV_mean_ normal cerebral cortex (no region specified)
Ikeda et al. (2013) [14]	SUV_mean_ tumor / SUV_mean_ contralateral frontal lobe (not specified if white or gray matter is measured)
Ryttlefors et al. (2016) [15]	Hotspot tumor / reference area in contralateral cortex including basal ganglia (hotspot is an area of 0.1 cm^3^ with the highest uptake in the meningioma ≈ comparable to SUV_peak_)
Mitamura et al. (2018) [25]	SUV_max_ tumor / SUV_max_ healthy frontal cortex, contralateral of the tumor SUV_peak_ tumor / SUV_peak_ healthy frontal cortex, contralateral of the tumor
Tomura et al. (2018) [8]	SUV_max_ tumor / SUV_max_ normal temporal lobe (not specified if white or gray matter is measured)
Jeltema et al. (2021) [current publication]	SUV_max_ tumor / SUV_max_ healthy contralateral (mirror) SUV_peak_ tumor / SUV_peak_ healthy contralateral (mirror) SUV_mean_ tumor / SUV_mean_ healthy contralateral (mirror) SUV_max_ tumor / SUV_max_ right parietal cortex (cortex) SUV_peak_ tumor / SUV_peak_ right parietal cortex (cortex) SUV_mean_ tumor / SUV_mean_ right parietal cortex (cortex)

### Analysis of imaging

The MRI/MET-PET report was made in collaboration by a nuclear physicist (AWJMG) and a neuroradiologist (AH) for all included patients. The MRI-only report was made by the neuroradiologist. To prevent a memory effect, there was a time interval of 6 weeks between the two reports for each patient. Also, the order in which the patient data had to be judged was altered, to prevent any familiarity with the diagnostic investigations.

### Statistical analysis

The SUV T/N ratios pre- and post-SRT were checked for significant differences using the Wilcoxon signed rank test. The agreement of the MET-PET/MRI report and the MRI-only report was calculated using Cohen’s κ. All statistical analysis was performed using IBM SPSS statistics version 23 (Chicago, IL, USA). A two-sided *P*-value < 0.05 was considered statistically significant.

## Results

### Patient characteristics

**Fig. 1:**
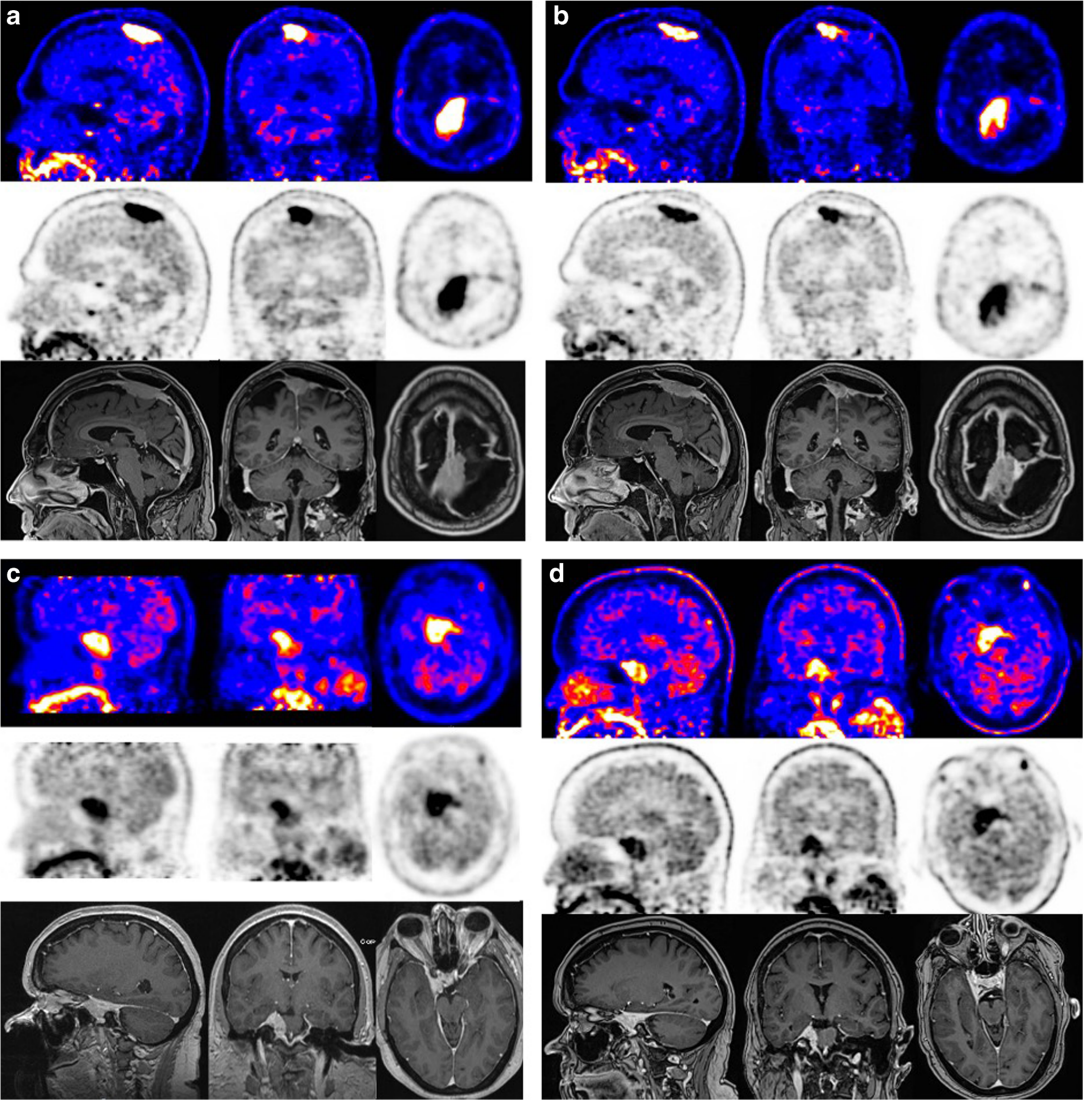
**a** Pre-SRT MET-PET and gadolinium-enhanced T1 MRI in a 62-year-old male patient, with previous partial resection of a parasagittal meningioma WHO grade I in September 2013. There is infiltration in the superior sagittal sinus. Because of growth of the tumor remnant during follow-up, SRT was installed in October 2017. **b** Post-SRT MET-PET and gadolinium-enhanced T1 MRI in the same, now 63-year-old patient, 15 months after SRT. MET-PET shows persistent raised ^11^C-methionine uptake in the tumor region. **c** Pre-SRT MET-PET and gadolinium-enhanced T1 MRI in a 63-year-old female patient with a meningioma extending in the right cavernous sinus. In December 2011, SRT was installed, without prior surgical resection. The treatment plan was made based on pathognomonic radiologic imaging. No tumor biopsy was performed. **d** Post-SRT MET-PET and gadolinium-enhanced T1 MRI in the same, now 70-year-old patient, 84 months after SRT. MET-PET shows persistent raised ^11^C-methionine uptake in the tumor region

Twenty patients participated in the study. The MET-PET examinations were performed between October 2018 and December 2019. Patient characteristics and tumor localizations are summarized in Table 2. The median age of the participants was 58.5 years (range 34–77). Twelve patients had one or more neurosurgical resections before the SRT. Most patients had a benign (WHO I) meningioma. One patient had an atypical (WHO II) meningioma. In two patients, the histopathological diagnosis was obtained with an ENT biopsy. In six patients, the decision for SRT was made based on pathognomonic radiological imaging. Fourteen patients were stereotactically irradiated with 54 Gy (30 fractions). The patient with the atypical meningioma received 60 Gy (30 fractions). Five patients were treated with a single-shot regimen of 14 Gy (four patients) or 20 Gy (one patient). The median time interval between SRT and the research MET-PET was 84 months (range 15–141). None of the included patients had radiological and/or clinical progression, necessitating a new treatment for the meningioma after SRT during follow-up. In retrospect, one patient with a benign meningioma had a pre-SRT MET-PET without ^11^C-methionine uptake. On the repeated MET-PET, SUVs were unchanged within normal range. The data of this patient were not used in the statistical analysis of the SUV uptake and T/N ratios. In the majority of patients, the post-SRT ^11^C-methionine uptake remained quantitatively and qualitatively remarkably high. Examples of the pre- and post-SRT MET-PETs and MRI scans of a patient with a parasagittal meningioma and a patient with a cavernous sinus meningioma are shown (Fig. 1).

**Table 2:** Patient characteristics (*n* = 20)

Characteristic	Value
Age in years, median (range)	58.5 (range 34–77)
Sex	
Male (%)	6 (30%)
Female (%)	14 (70%)
Localization of meningioma	
Parasagittal	3 (15%)
Cavernous sinus	4 (20%)
Bifrontal skull base	1 (5%)
Tuberculum sellae	2 (10%)
Cerebellopontine angle (CPA)	1 (5%)
Petroclival	2 (10%)
Clivus	1 (5%)
Sphenoidal	1 (5%)
High cervical level (C2/3 level)	1 (5%)
Tentorial	4 (20%)
Surgical resection before SRT (%)	12 (60%)
Months after radiotherapy, median (range)	84.0 (15–141)
Patients with progressive disease^a^	0 (0%)
Histological grade	
WHO I (%)	13 (65%)
WHO II (%)	1 (5%)
WHO III (%)	0
No histology/unknown (%)	6 (30%)

^a^Defined as the necessity to start a new line of oncologic treatment after SRT (e.g., re-irradiation, re-resection, systemic treatment) or change of the treatment goal to best supportive care

### MET-PET SUV in meningiomas after SRT

T/N ratios were calculated for SUV_max_, SUV_peak_, and SUV_mean_ for nineteen patients_._ Ratios for tumor-to-contralateral-healthy (mirror) and tumor-to-right-parietal-healthy (cortex) were determined (Table 3). For each of these values, the post-SRT ^11^C-methionine uptake in the tumor region was increased at least twofold (range 2.16–3.17). In fourteen patients, the post-SRT MET-PET could be compared to the pre-SRT MET-PET (Table 4). The median duration of follow-up in this subgroup was 79 months (range 15–90). In the remaining five patients, the pre-SRT MET-PET was not saved in a format that allowed calculation of SUVs with the current software. There was no statistical difference between the pre-and post-SRT ^11^C-methionine uptake for five different categories of SUV T/N ratios (SUV_max_ T/N_mirror_, SUV_max_ T/N_cortex_, SUV_peak_ T/N_mirror_, SUV_mean_ T/N_mirror_, and SUV_mean_ T/N_cortex_). Only the SUV_peak_ T/N_cortex_ decreased significantly from 2.57 (SD1.02) to 2.20 (SD0.87) (*p* = 0.004).

**Table 3:** Mean post-SRT T/N ratios for the different MET-PET SUV values (*n* = 19)

T/N ratio	Mean (SD)
SUV_max_ T/N_mirror_	3.17 (1.31)
SUV_peak_ T/N_mirror_	2.51 (0.79)
SUV_mean_ T/N_mirror_	3.07 (1.30)
SUV_max_ T/N_cortex_	2.42 (0.81)
SUV_peak_ T/N_cortex_	2.16 (0.84)
SUV_mean_ T/N_cortex_	2.39 (0.76)

**Table 4:** Comparison of pre-SRT MET-PET and post-SRT MET-PET (*n* = 14)

	Pre-SRT mean (SD)	Post-SRT mean (SD)	*p*
SUV_max_ T/N_mirror_	2.80 (0.91)	2.92 (0.76)	0.93
SUV_peak_ T/N_mirror_	2.64 (0.92)	2.51 (0.69)	0.20
SUV_mean_ T/N_mirror_	2.81 (0.86)	2.99 (1.19)	0.78
SUV_max_ T/N_cortex_	2.47 (0.87)	2.33 (0.75)	0.33
SUV_peak_ T/N_cortex_	2.57 (1.02)	2.20 (0.87)	**0.004**
SUV_mean_ T/N_cortex_	2.64 (0.88)	2.35 (0.73)	0.13

### MRI-only versus combined MRI/MET-PET-report

Agreement between the MRI-only report and the MRI/MET-PET report was calculated with Cohen’s kappa. The MRI-only and the MRI/MET-PET report were judged as “progressive disease” or “not progressive disease.” There was a good agreement concerning this outcome with a kappa of 0.77. In only one patient, the reports were not congruent. In this case, the combined MET-PET/MRI report concluded progressive disease (PD), while the MRI-only report concluded stable disease (SD).

## Discussion

Nuclear imaging is an informative and often complementary imaging technique in the oncologic follow-up of many types of neoplasms. We evaluated MET-PET in the follow-up of meningioma patients after SRT. First, we found that ^11^C-methionine uptake remained (both qualitatively and quantitatively) remarkably high after SRT in patients with stable disease. Previous literature on the topic of ^11^C-methionine uptake in meningiomas also shows persistent raised uptake ratios in meningioma tumor tissue. In our pilot study, there was no robust decrease in ^11^C-methionine uptake after a median follow-up period of more than 6 years after SRT. Second, the κ-coefficient of the MRI-only report and the combined MET-PET/MRI report had a good agreement, demonstrating that additional MET-PET did not change the interpretation of regular MRI follow-up in this pilot study. Hence, the added value of MET-PET in the follow-up of stereotactically irradiated meningiomas could not be substantiated based on these data.

We compared our results to several other previous studies on the use of MET-PET in the follow-up of meningioma patients. In general, a striking difference in the methodology of analyzing MET-PET scans was encountered (Table 1.). Ikeda et al. included 37 meningioma patients in a MET-PET follow-up study. The majority of their patients had a previous neurosurgical resection; some patients were on a wait-and-scan policy. There were no patients with an irradiated meningioma in their population. They reported eight tumor recurrences, which had significantly higher MET-PET T/N ratios than the stable tumors. The group with PD had a T/N ratio of 3.84 ± 1.13, and the group with SD had a T/N ratio of 2.74 ± 1.02 [*p* < 0.01]. Based on receiver-operating characteristic (ROC) analysis, they found an optimal cutoff at a T/N ratio of 3.18 (sensitivity 63%; specificity 79%) [14]. In this study, the evolvement of MET-PET scans of meningioma patients over time was not analyzed. In the study of Ryttlefors et al., nineteen meningioma patients treated with proton beam therapy had a MET-PET in adjunct to their regular follow-up MRI during a period of 10 years after treatment [15]. Two patients had disease progression during follow-up. Overall, there was a significant decrease in ^11^C-methionine uptake during this long follow-up period. They found, in the subgroup of patients with 10 years of follow-up, a decrease from an initial T/N ratio of 4.7 to a T/N ratio of 3.4 (*p* < 0.01). The change in T/N ratio was significantly larger in patients with PD than in patients with SD after 7 years of follow-up, with ratios of 1.36 and 0.77 (*p* < 0.01) respectively. The authors explicitly mention that the interpretation of MET-PET in the follow-up of meningiomas is not straightforward. They encountered several patients with a significant increase of ^11^C-methionine uptake in the first years, before decreasing in uptake at 10 years of follow-up. They also encountered multiple patients with a so-called erratic uptake pattern, which showed multiple ^11^C-methionine uptake rises and decreases during the 10 years of follow-up, without a clear clinical correlate. Ryttlefors et al. conclude that MET-PET can be used as an adjunct but not as a replacement of MRI in the follow-up of meningioma patients. When compared to our data, it must be noted that a different radiation modality is applied in this study, with a different treatment protocol. The number of included patients in our pilot study is fairly low to allow for further subgroup analysis. However, in our data, we found no significant difference between patients with a follow-up duration of < 5 years and ≥ 5 years, regarding T/N ratio difference pre- and post-SRT. Also, no correlation was found between the duration of follow-up and the change in T/N ratio over time. Comparing both the abovementioned studies, it is striking that the uptake ratios in the series of Ryttlefors et al., including the ratios of the patients with stable disease, were all above the cutoff value of 3.18 found by Ikeda et al.

The uptake ratios of ^11^C-methionine are even higher in the retrospective series of Mitamura et al. In 22 new meningioma patients, they found a mean SUV_max_ T/N ratio of 5.32 and a mean SUV_peak_ T/N ratio of 4.05, together with a statistically significant higher uptake in WHO grade II tumors compared to WHO grade I tumors (*p* = 0.002) [25]. Tomura et al. found a mean SUV_max_ T/N ratio of 3.24 ± 1.36 in their study population of seventeen patients with grade II or III meningiomas [8]. Lastly, Arita et al. found a SUV_mean_ T/N ratio of 2.20 and a SUV_max_ T/N ratio of 4.09 for skull base meningiomas, which was significantly higher than the uptake T/N ratios for non-skull base meningiomas [24]. In these three studies, no comparison of MET-PET results at different time points during follow-up was performed, as was done in our pilot study.

An important conclusion from the stated literature is that the reported data and methodology are very “center-specific” and not suitable for extrapolation to other clinical settings. Concerning methodology, it is very important to notice that all studies employ a different method to determine the T/N ratio. Also, different acquisition parameters and PET cameras were used. We summarize the diversity of calculating T/N ratios for the above cited articles in Table 1. Consequently, there is a clear need for a standardized approach of determining T/N ratios in meningioma PET scanning. Also, the choice of type of SUV value needs to be uniform. Currently, in nuclear medicine, SUV_peak_ is most preferred. SUV_mean_ is arguably the most subjective and unreliable outcome parameter, because its value is greatly dependent on the volume-of-interest (VOI) that is being chosen. In our pilot study we used an ellipsoid VOI, in which the tumor region was captured. We did not have the software to perform the calculation of SUV_mean_ using an intensity based VOI, in which the isocontour is a percentage of SUV_max_. The latter technique is informative on the metabolically active tumor volume (and would ideally be related to the baseline and follow-up MRI tumor volume).

In our study, three different types of SUV values were evaluated (SUV_max_, SUV_peak_, and SUV_mean_). Also, two types of reference regions were used (contralateral/mirror and right parietal/cortex) for the calculation of T/N ratios. The aim of this multivariable outcome analysis was to avoid an outcome that is very “protocol-specific.” Considering the outcome values, we did not find a robust decrease in ^11^C-methionine uptake in our population after SRT. An explanation why the SUV_peak_ T/N_cortex_ was the only outcome parameter out of six, which showed a significant decrease after SRT, is not easy to give. However, it is important to realize that the other five outcome parameters did not decrease significantly and that two actually showed a nonsignificant increase over time. SUV_max_ and SUV_mean_ are more often employed in the literature on meningioma PET scanning than SUV_peak_. Besides that, the visual qualitative aspect of MET-PET uptake remained very high. Therefore, we conclude that ^11^C-methionine amino acid metabolism remains too high after SRT in meningiomas, for allowing MET-PET to be a useful add-on to the regular MRI follow-up.

This renders the question whether there are other promising PET modalities for the follow-up of meningiomas. Tracers directed to the somatostatin/SSTR2a receptor seem the most promising, since the majority of meningiomas express this receptor. ^68^Ga-DOTATATE and ^68^Ga-DOTATOC PET are examples of nuclear imaging techniques that make use of a tracer directed to the somatostatin receptor [17, 26]. Ivanidze et al. show an excellent differentiation between viable meningioma and treatment-induced changes in their series of twenty patients, in which ^68^Ga-DOTATATE PET was applied as an add-on to MRI to recognize tumor recurrence/progression, in patients who underwent surgical resection or radiotherapy previously [17]. A disadvantage of the technique in a small subset of patients is that the pituitary gland also shows a high uptake of this tracer and often serves as a positive control. This might influence the diagnostic gain for skull base lesions in close proximity to the pituitary gland. In daily clinical practice, this is probably true for only a minority of the cases. In their review, Galldiks et al. mention a role for PET ligands to the SSTR2a receptor in target delineation for surgical resection and radiotherapy and the differentiation of tumor progression from post-therapeutic effects in meningioma patients [7]. Rachinger et al. correlated ^68^Ga-DOTATATE uptake to the histopathologic analysis of tumor specimens and healthy surrounding tissue in 21 patients. They found a sensitivity of 90.1% and a specificity of 73.5% for ^68^Ga-DOTATATE PET regarding discernment of tumor from tumor-free tissue, which was higher than could be achieved with contrast-enhanced MRI. ROC analysis revealed a SUV_max_ of 2.3 as optimal cutoff [16]. This series included only nine patients with recurrent tumors, of which only two had a form of radiotherapy (Cyberknife) as part of their meningioma treatment. Therefore, it is difficult to extrapolate these data to (stereotactically) irradiated meningiomas. This is emphasized by the fact that in this study, six out of nine recurrent tumors did have no positive or only weak SSTR2 immunohistochemical staining. Hence, there is a clear need for validation of this technique in larger, multicenter trials. The effect of adding PET imaging to the regular MRI follow-up of meningioma patients (for both MET-PET and SSTR2a ligand PET) on treatment outcome has not been evaluated.

### Study limitations

Patients without or with limited neurological symptoms and stable disease are considered to be motivated to participate in a prospective pilot study like this, whereas invalidated patients with disease progression will less likely opt for study participation. Also, MET-PET study data of deceased patients is not available. This results in a potential selection bias. This is a pilot study with a small number of patients. Not for every included patient, the pre-SRT MET-PET was available for analysis (the pre-SRT MET-PET was available in fourteen out of twenty patients). There was a notable range in the follow-up duration of the patients in this study. Results should be confirmed in a larger cohort of meningioma patients, preferably in a multicenter setting.

## Conclusions

We report remarkably high quantitative and qualitative ^11^C-methionine uptake T/N ratios after SRT, in patients with a meningioma of the skull base or close to large vascular structures. Additional MET-PET to the regular MRI follow-up had no impact on the interpretation of follow-up imaging in this series.

Also, there is a notable lack of standardized outcome measurement concerning this type of nuclear investigation in meningioma patients. Possibly other PET-modalities, e.g., ^68^Ga-DOTATATE and ^68^Ga-DOTATOC PET, are more informative in this patient category with often difficult to interpret follow-up MRI after SRT.

## Abbreviations

CPA: Cerebellopontine angle
FDG-PET: Fluorodeoxyglucose positron emission tomography
FET-PET: ^18^F-Fluoroethyl-L-tyrosine positron emission tomography
L/N: Lesion-to-normal
MET-PET: ^11^C-Methionine positron emission tomography
MRI: Magnetic resonance imaging
PD: Progressive disease
PET: Positron emission tomography
ROC: Receiver-operating characteristic
SD: Stable disease
SRT: Stereotactic radiotherapy
SUV: Standardized uptake value
T/N: Tumor-to-normal
VOI: Volume-of-interest
